# Neurological graft-versus-host disease with MOG antibody positivity after allogeneic stem cell transplantation: a case report

**DOI:** 10.3389/fimmu.2026.1684838

**Published:** 2026-03-11

**Authors:** Xin Zhou, Ningxia Song, Zhaohong Xie, Ai Li, Juandong Wang, Ningning Shan

**Affiliations:** 1 Department of Hematology, The Second Qilu Hospital of Shandong University, Jinan, Shandong, China; 2 Department of Neurology, The Second Qilu Hospital of Shandong University, Jinan, Shandong, China

**Keywords:** antibody, CNS demyelination, haploidentical stem cell transplantation, live-cell immunofluorescence, MOGAD

## Abstract

**Background:**

Central nervous system (CNS) involvement is recognized as a distinct manifestation of chronic GVHD (cGVHD). Myelin oligodendrocyte glycoprotein antibody–associated disease (MOGAD) is an autoimmune disorder targeting the CNS. Therefore, the occasional detection of MOG antibodies in patients with CNS-cGVHD may represent a manifestation within the spectrum of CNS-GVHD.

**Case presentation:**

A 22-year-old female with severe aplastic anemia (AA) underwent maternal haploidentical hematopoietic stem cell transplantation (HSCT) in May 2021. During conditioning, she developed acute neurotoxicity. Subsequent complications included acute and chronic GVHD as well as posterior reversible encephalopathy syndrome (PRES). Three months after transplantation, she developed sensory deficits and ataxia, and cervical magnetic resonance imaging revealed inflammatory lesions consistent with cervical myelitis. Comprehensive neuroimmunological evaluation excluded alternative infectious, metabolic, or neoplastic etiologies. Anti–myelin oligodendrocyte glycoprotein (MOG) antibodies were negative by cell-based assay (CBA) and tissue-based assay (TBA), but demonstrated weak, low-titer positivity on live-cell immunofluorescence. Neurological function recovered following treatment with intravenous immunoglobulin (IVIG) and methylprednisolone.

**Conclusions:**

This case illustrates low-titer post-HSCT MOG seropositivity detected only by a live cell–based assay in a patient who fulfilled diagnostic criteria for CNS-GVHD. Live cell–based MOG testing may be considered in selected HSCT recipients with unexplained or atypical neuroinflammatory syndromes despite negative conventional assays, with results interpreted cautiously as hypothesis-generating for individualized immunosuppressive management.

## Introduction

Aplastic anemia (AA) is a bone marrow failure syndrome, for patients with confirmed AA, primary treatment options are allogeneic hematopoietic stem cell transplantation (HSCT) or immunosuppressive therapy, with current long-term survival rates exceeding 70% (1, 2). Graft-versus-host disease (GVHD), a major and potentially life-threatening complication of allogeneic HSCT (3), is classified as acute or chronic based on distinct clinical manifestations and pathophysiological mechanisms (4, 5). Historically, the CNS was considered relatively protected from GVHD by the blood-brain barrier (BBB). However, accumulating evidence from clinical research and case reports of GVHD patients with neurological symptoms has established CNS involvement as a distinct manifestation of cGVHD, as recognized in the consensus criteria from the Clinical Practice Conference on cGVHD (6). Myelin oligodendrocyte glycoprotein antibody-associated disease (MOGAD) is an autoimmune demyelinating disorder. Characterized by antibodies targeting MOG, it predominantly affects the optic nerves and spinal cord, typically presenting with vision loss and paralysis (7). Current epidemiological data indicate that MOGAD primarily affects young Caucasian populations, with a lower female predominance compared to other autoimmune disorders of the CNS (8). In Chinese cohorts, 20.7% of MOG-seropositive patients develop typical encephalitic manifestations during disease progression (9). Recent population-based studies from Europe, Asia, and the Americas suggest that MOGAD is a rare disorder with a global prevalence of approximately 1.3–2.5 per 100,000 and an annual incidence of about 1.6–4.8 per million people, with broadly similar rates across regions. These data indicate that, although many early cohorts originated from Asian centers, MOGAD is now recognized worldwide as an uncommon but clinically important cause of CNS demyelination (10, 11). This case suggests that transient low-titer MOG antibody reactivity may occur in the context of CNS-GVHD after allo-HSCT and may represent a bystander autoimmune phenomenon related to CNS-GVHD rather than definitive MOGAD.

## Case presentation

We report a 22-year-old woman with an 11-year history of severe aplastic anemia who had shown no meaningful response to cyclosporine, androgens, or hematopoietic growth factor therapy. She was admitted on April 26, 2021, for maternal haploidentical hematopoietic stem cell transplantation (Haplo-HSCT), based on an 8/12 HLA match with her mother. A conditioning regimen was administered, consisting of busulfan (0.8mg/kg q6h, days -7 to -6), fludarabine (30mg/m^2^ daily, days -7 to -4), cyclophosphamide (40mg/kg daily, days -5 to -3), and antithymocyte globulin (2.5mg/kg daily, days -5 to -2), alongside cyclosporine, mycophenolate mofetil, and short-course methotrexate for GVHD prophylaxis.

On May 6 (day -5), the patient developed sudden severe occipital headache, dizziness, visual disturbances, and nausea, prompting immediate discontinuation of intravenous cyclosporine. After administering 40 mg methylprednisolone IV, symptoms improved. One hour later, the patient had a generalized tonic-clonic seizure, treated with 10 mg diazepam IV, stopping the seizure. MRI was not performed due to severe bone marrow suppression. On May 9 (day −2), the patient reported blurred vision and occipital pain, accompanied by right upper limb weakness, with muscle strength graded as IV on physical examination. Brain CT revealed multiple small ischemic infarcts in the bilateral occipital lobes, left basal ganglia, and bilateral parietal lobes. Following neurological consultation, acute ischemic stroke was considered. Given the patient’s thrombocytopenia, anticoagulant and antiplatelet therapies were contraindicated; therefore, neuroprotective and anti-edema therapy with butylphthalide and mannitol (150 mL every 12 hours) was initiated. After symptomatic treatment, muscle strength of the right upper limb decreased to grade III, and the patient reported improvement in visual disturbance.

By May 10, after completion of the conditioning regimen, the patient reported persistent blurred vision and inability to hold objects with the right upper limb. Following exogenous platelet transfusion, the platelet count increased to 87 × 10^9^/L. After neurological consultation and in accordance with relevant treatment guidelines (12, 13), and based on her platelet level and clinical status, she was treated with enoxaparin (30 mg), citicoline sodium, edaravone, and intravenous fluids. On May 11 and 12, her visual disturbance showed slight improvement, while muscle strength remained unchanged. During this period, she received two infusions of donor-derived hematopoietic stem cells.

**Figure 1:**
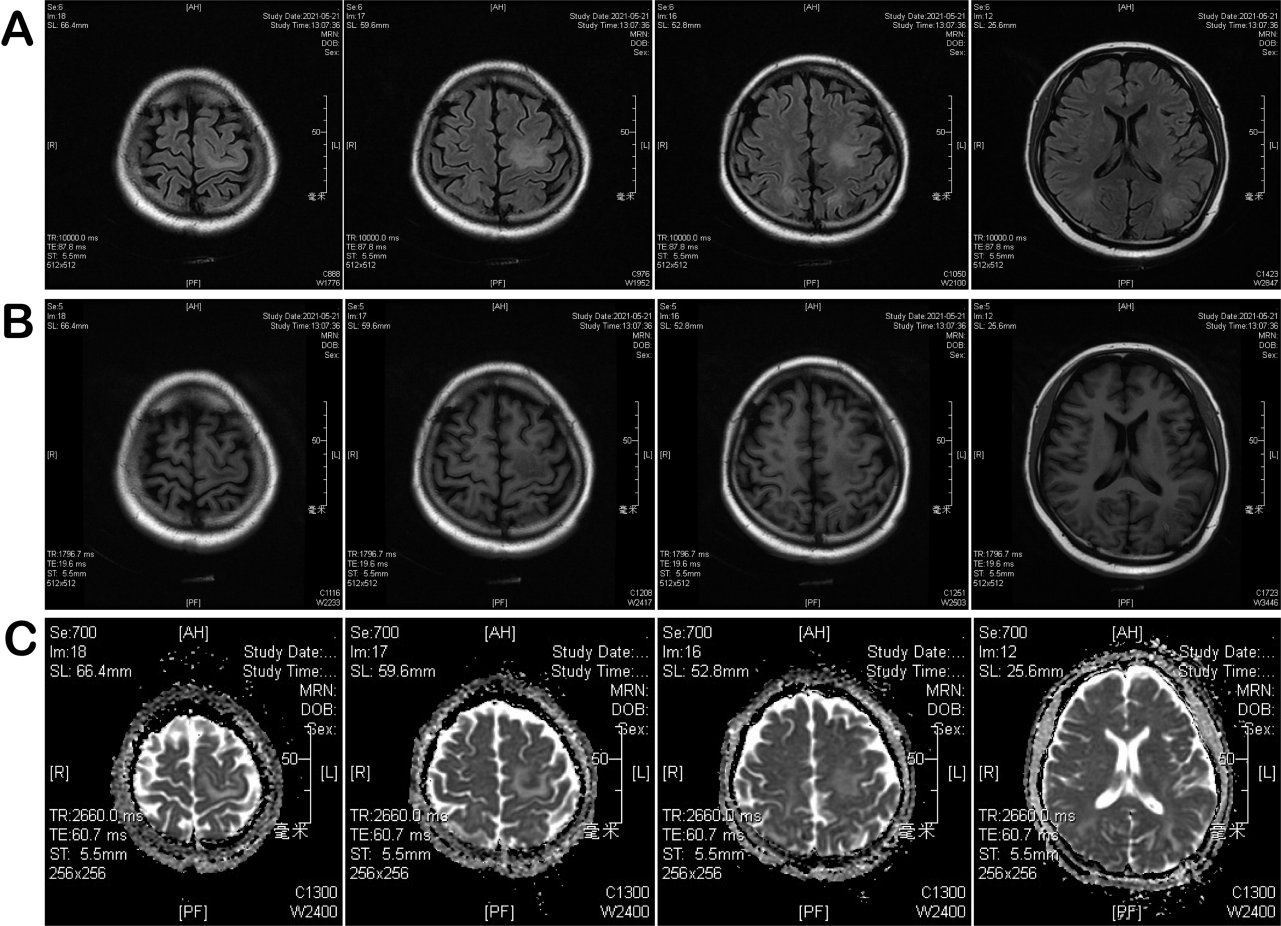
MRI performed on May 21, 2021: **(A)** Brain/spinal fluid-attenuated inversion recovery (FLAIR) sequence; **(B)** T1-weighted gadolinium-enhanced sequence; **(C)** diffusion-weighted imaging and apparent diffusion coefficient (DWI-ADC) maps.

From May 13 to May 19, the patient’s vision basically returned to normal, and her fine motor coordination and right upper-limb muscle strength gradually improved, accompanied by hematopoietic recovery. However, on May 21 (day +10), the patient complained of lower lip numbness and occipital pain. Brain MRI revealed multiple intracranial abnormal signal lesions, predominantly symmetric and mainly involving the posterior regions. Scattered patchy and strip-like long T1 and T2 signal lesions were observed in the bilateral frontal cortex, parietal-occipital lobes, and bilateral corona radiata of the basal ganglia. These lesions appeared hyperintense on FLAIR, showed focal hyperintensity on DWI in the frontal and parietal-occipital regions, and did not demonstrate low signal on ADC maps. In the context of a history of hematopoietic stem cell transplantation and immunosuppressive therapy, these findings were considered highly suggestive of PRES (**Figures 1A–C**) (**Table 1**). Potential contributing factors, including calcineurin inhibitor–related neurotoxicity and GVHD-associated immune-mediated injury, were considered. Accordingly, intravenous cyclosporine was switched to oral tacrolimus with maintenance of a low therapeutic trough level, and methylprednisolone was added.

**Table 1:** Imaging summary table.

Time	Symptoms	Imaging type	Imaging findings
May 9, 2021	blurred vision, occipital pain	CT	Multiple small ischemic infarcts in bilateral occipital lobes, left basal ganglia, bilateral parietal lobes
May 21, 2021	Lip numbness, occipital pain	MRI	Scattered long T1/T2 lesions; high DWI signal without ADC decrease; microhemorrhages on SWAN; normal large vessels on MRA; reduced perfusion in left cerebral hemisphere and right cerebellum
August 17, 2021	Reduced temperature and pain sensation; sensory ataxia	CT	Slight low-density area in left basal ganglia radiating crown
August 17, 2021	Same as above	Brain MRI	Right-sided acute/subacute infarction in pons, abnormal signals suggesting PRES; long T1 lesion in occipital sulcus; multiple microbleeds on SWAN; reduced perfusion on ASL
August 17, 2021	Same as above	Cervical MRI	Abnormal anterior cord signal at C1–2 suggesting inflammatory injury; C5/6 and C6/7disc herniation with mild stenosis
November 24, 2021	Neurological symptoms improving	Brain MRI + DWI	Decreased signal intensity and reduced volume of multiple lesions involving left basal ganglia, bilateral corona radiata, bilateral frontoparietal–occipital regions, and left corpus callosum; some DWI hyperintense lesions less conspicuous; prior cortical lesions no longer visualized
November 24, 2021	Same as above	Cervical MRI	Scattered patchy cervical cord lesions with reduced extent, indicating interval radiological improvement
August 14, 2022	Asymptomatic/stable	Brain MRI + DWI	Only a few small punctate residual lesions in bilateral corona radiata; no diffusion restriction; ventricular system unremarkable
August 14, 2022	Same as above	Cervical MRI	No definite focal abnormal cervical cord signal, stable to improved compared with prior

On May 25 (day +14), the patient developed skin erythema primarily affecting the face, neck, anterior chest, back, and palms, consistent with acute grade 2 GVHD (stage I), and the lower lip numbness had improved compared with before. GVHD risk factor testing indicated a high likelihood of severe aGVHD (**Supplementary Figure 1**), and she was treated with 1 mg/kg of methylprednisolone. After three days of treatment, the skin rash progressively worsened, with expansion of the affected area and the appearance of localized vesicles. Concurrently, the patient developed grade 1 gastrointestinal aGVHD, which subsequently progressed to grade II. Due to an inadequate response to corticosteroids, treatment was intensified with baliximab, ruxolitinib, and umbilical cord MSCs, specifically: baliximab 20 mg administered on days +1, +3, and +8; ruxolitinib 5 mg twice daily, increased to 10 mg twice daily after three days; and umbilical cord MSCs at 1 × 10^6^/kg twice weekly for four doses. On day 25 of treatment, the patient developed severe cutaneous aGVHD. After consultation with dermatology, recombinant human TNF receptor type II fusion protein (etanercept) was added to the treatment regimen at a dose of 25 mg administered intrahypodermically for two consecutive days. During follow-up, the patient developed mild chronic GVHD involving the skin (Score 1)(**Supplementary Figure 2**), oral mucosa, and nails, which improved with tacrolimus, ruxolitinib, and low-dose corticosteroids. At that time, the numbness of the lower lip had improved compared with before, neurological symptoms were alleviated, and PRES was well controlled.

**Figure 2:**
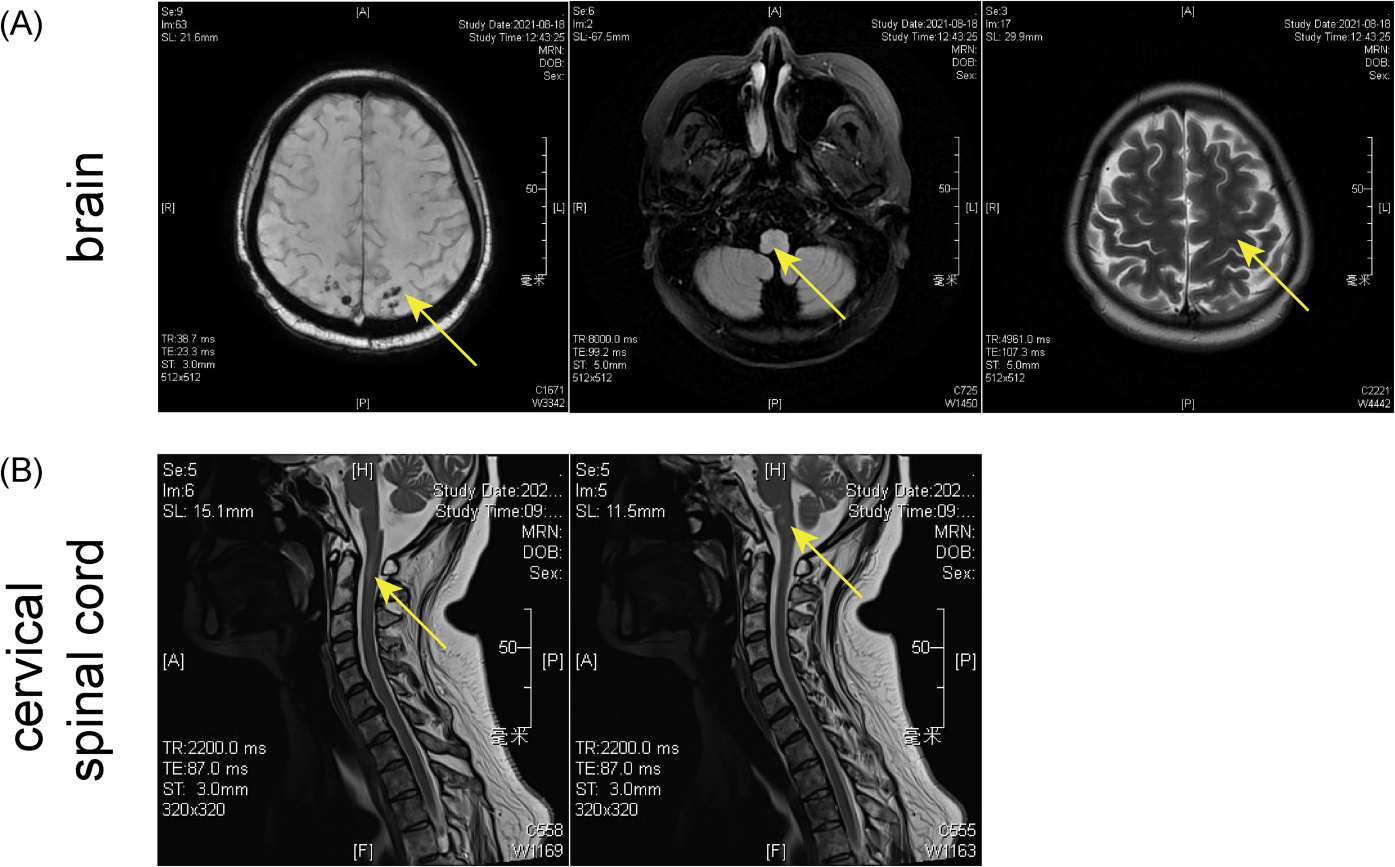
MRI imaging of the brain **(A)** and cervical spine **(B)** 3 months after haplo-HSCT treatment. This figure illustrates the dynamic radiographic evolution of CNS lesions across multiple time points following HSCT. Early imaging demonstrated scattered cortical–subcortical T2/FLAIR hyperintensities with minimal diffusion restriction, initially suggestive of PRES. Subsequent MRI studies revealed progression to multifocal white matter involvement, microhemorrhages on SWI, and new patchy lesions in the brainstem and cervical spinal cord, findings that were not fully consistent with isolated PRES. Later imaging showed partial resolution of vasogenic edema but emergence of immune-mediated demyelinating features, correlating with clinical deterioration and the development of systemic chronic GVHD.

**Figure 3:**
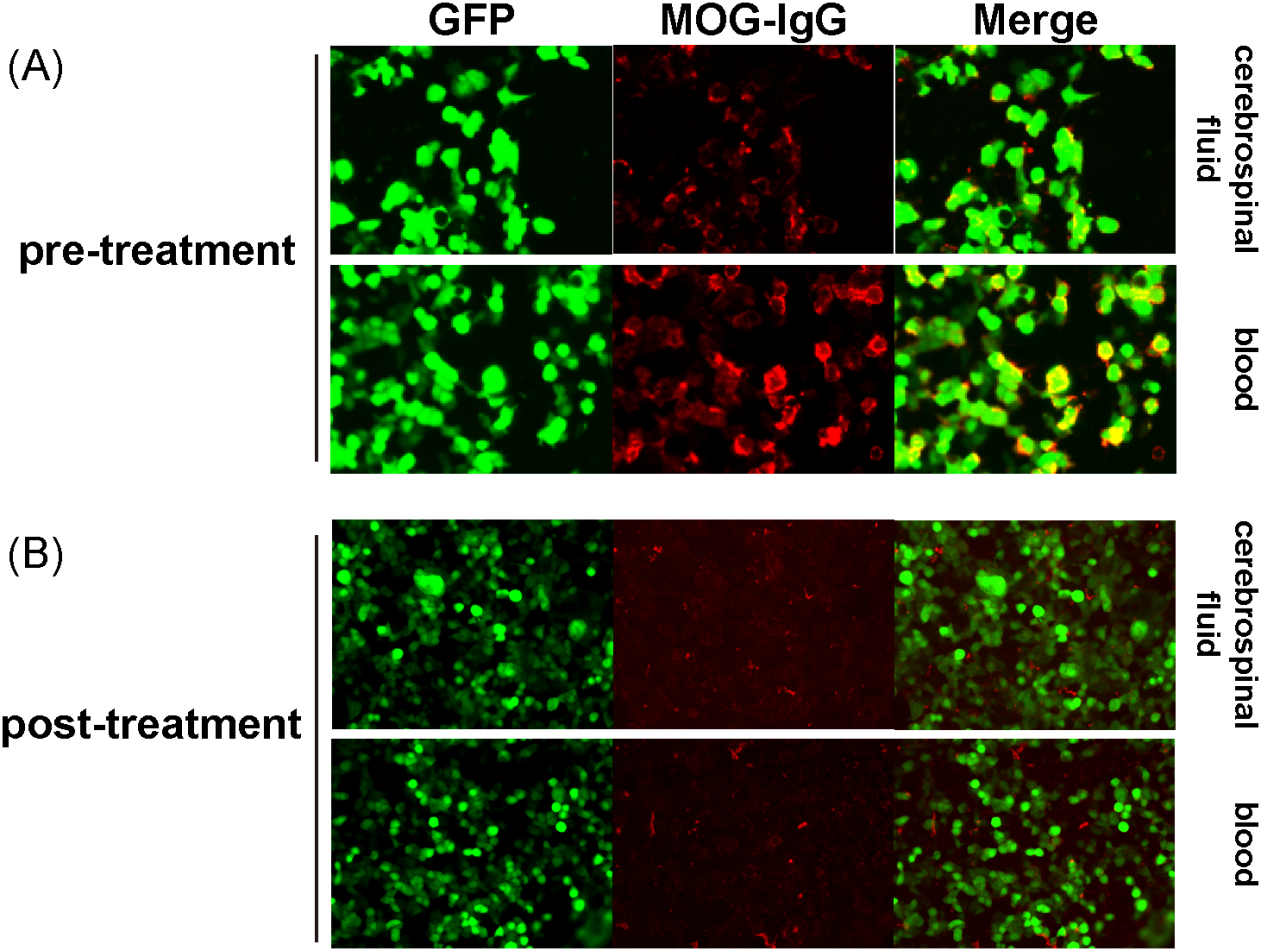
Live Cell Immunofluorescence Detection of MOG Antibodies 3 Months After Haplo-HSCT Treatment. **(A)** Before Treatment with Immunoglobulin and Methylprednisolone. **(B)** After Treatment with Immunoglobulin and Methylprednisolone. The green light represents live cells stained with GFP, and the red light represents immunoglobulin G (IgG) bound to MOG.

Three months after transplantation, on August 17, 2021, she also experienced reduced temperature and pain sensation and sensory ataxia in the right upper and lower limbs. Head CT revealed a slight low-density area in the left basal ganglia radiating crown region. Brain MRI showed right-sided acute/subacute brain infarction in the pons, multiple abnormal signals in the brain, a long T1 signal lesion in the occipital sulcus, multiple low signals on SWAN, and reduced perfusion in the left cerebral hemisphere on ASL (**Figure 2A**). Cervical MRI showed abnormal anterior spinal cord signals at the C1–2 level, suspected inflammatory damage, along with disc herniation at C5/6 and C6/7, and mild spinal stenosis (**Figure 2B**). Based on cranial imaging findings together with blood and cerebrospinal fluid analyses, infectious, metabolic, cerebrovascular, neoplastic, and drug-related central nervous system complications were excluded, raising suspicion for autoimmune-mediated neurogenic injury. (The cerebrospinal fluid examination results are summarized in **Table 2**). According to the recommendations from the multidisciplinary consultation, testing for MOG-related antibodies was advised. Although MOG antibodies were negative by cell-based assay (CBA) and tissue-based assay (TBA) (**Supplementary Figure 3**), weakly positive result (1:32 dilution) were detected using live cell immunofluorescence (**Figure 3A**). The detailed procedure for the live cell–based assay is provided in the **Supplementary materials**. A preliminary diagnosis of immune-mediated CNS injury was made, and treatment with intravenous immunoglobulin (0.4 g/kg for 5 days) and methylprednisolone (40 mg) led to rapid relief of hyperalgesia in the right limbs and serum conversion of MOG antibodies (**Figure 3B**).

**Table 2:** Cerebrospinal fluid examination.

Item	Result	Method/type
Folate	2.62 ng/mL	Biochemical
Vitamin B12	Normal	Biochemical
White blood cell count	2 × 10^6^/L	Routine
Protein	Normal	Biochemical
Chloride	Normal	Biochemical
Glucose	5.37 mmol/L	Biochemical
Lactate	2.6 mmol/L	Biochemical
Cytology	Scant lymphocytes	Cytological
NMDA receptor antibody IgG	Negative	CBA
AMPA1 receptor antibody IgG	Negative	CBA
AMPA2 receptor antibody IgG	Negative	CBA
LGI1 antibody IgG	Negative	CBA
GABAB receptor antibody IgG	Negative	CBA
CASPR2 antibody IgG	Negative	CBA
IgLON5 antibody IgG	Negative	CBA
DPPX antibody IgG	Negative	CBA
GlyR1 antibody IgG	Negative	CBA
DRD2 antibody IgG	Negative	CBA
GAD65 antibody IgG	Negative	CBA
mGluR5 antibody IgG	Negative	CBA
Aquaporin-4 (AQP4) antibody	Negative	CBA
Myelin oligodendrocyte glycoprotein (MOG) antibody	Negative	CBA
Glial fibrillary acidic protein (GFAP) antibody	Negative	CBA
Myelin basic protein (MBP) antibody	Negative	CBA
Cytomegalovirus (CMV) antibody	Negative	Immunoassay
Herpes simplex virus type 1 (HSV-1) antibody	Negative	Immunoassay
Herpes simplex virus type 2 (HSV-2) antibody	Negative	Immunoassay
Epstein–Barr virus (EBV) antibody	Negative	Immunoassay
Parainfluenza virus antibody	Negative	Immunoassay
Varicella-zoster virus (VZV) antibody	Negative	Immunoassay
Viral serology panel	Negative	Immunoassay

**Figure 4:**
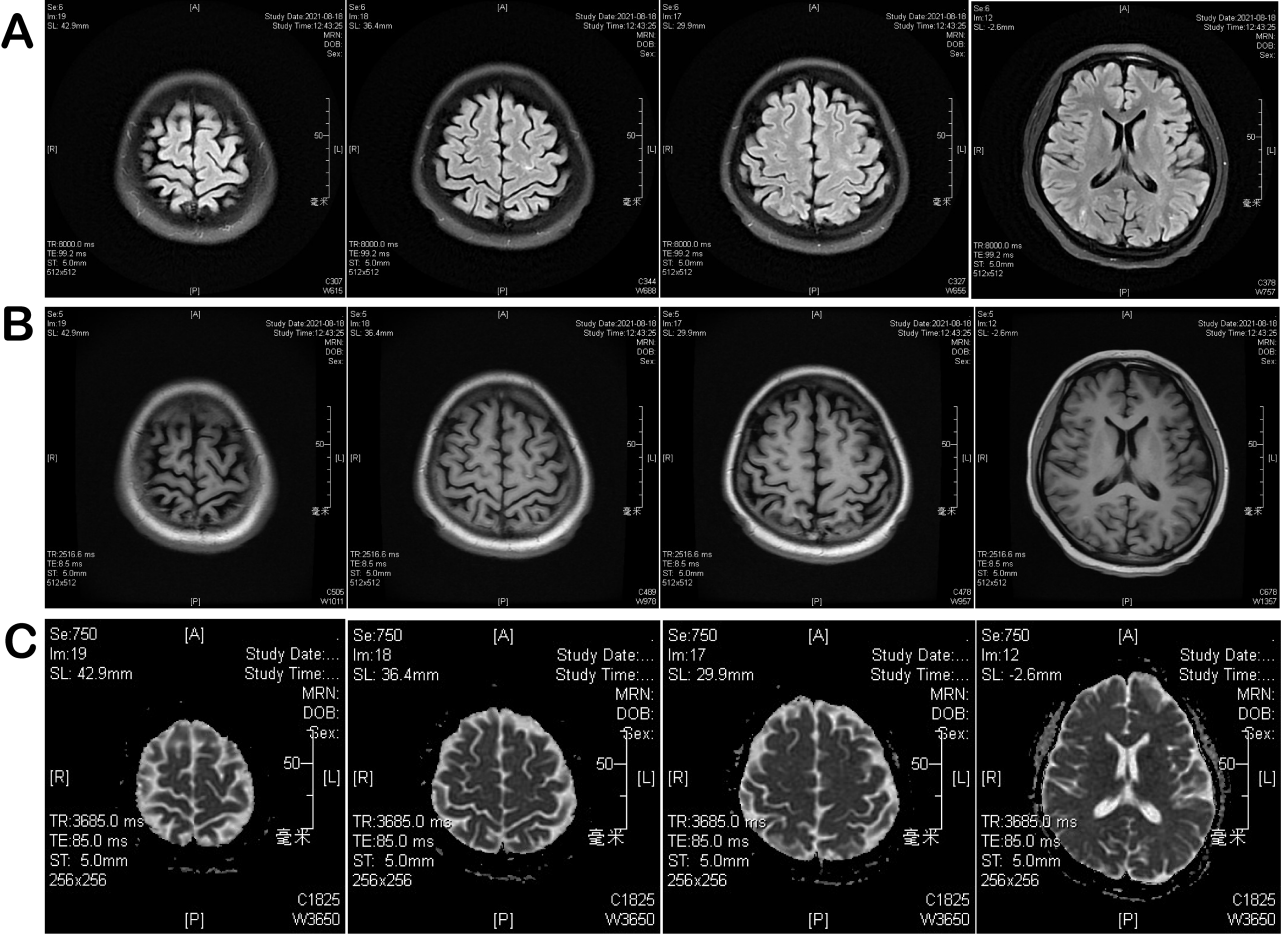
MRI performed on August 14, 2022: **(A)** Brain/spinal fluid-attenuated inversion recovery (FLAIR) sequence; **(B)** T1-weighted gadolinium-enhanced sequence; **(C)** diffusion-weighted imaging and apparent diffusion coefficient (DWI-ADC) maps.

Follow-up brain MRI with DWI and cervical spinal MRI on November 24, 2021 demonstrated decreased signal intensity and reduced volume of multiple lesions involving the left basal ganglia, bilateral corona radiata, bilateral frontoparietal–occipital regions, and the left body of the corpus callosum compared with prior imaging. Some previously noted DWI hyperintense lesions became less conspicuous. Linear cortical lesions in the left frontal lobe and bilateral occipital lobes were no longer visualized, and subcortical abnormal signals in the right cerebral hemisphere had partially resolved. Scattered patchy lesions within the cervical spinal cord showed a reduced extent, indicating interval radiological improvement (**Supplementary Figure 4A**). Follow-up MRI on August 14, 2022 revealed a small nodular lesion with long T1 and T2 signal intensity in the right pons, showing marked hyperintensity on DWI. Scattered punctate long T1 and T2 signal lesions were observed in the bilateral frontal cortex, parietal-occipital lobes, and bilateral corona radiata of the basal ganglia, appearing hyperintense on FLAIR without obvious hyperintensity on DWI, with a markedly reduced extent compared with the previous MRI on May 21, 2021(**Figures 4A–C**). In addition, a linear long T1 signal lesion was noted in the occipital sulcus. The ventricular system was unremarkable. No definite focal abnormal signal was identified within the cervical spinal cord, consistent with stable to improved spinal cord findings (**Supplementary Figure 4B**). At present, the patient has achieved complete neurological recovery and maintains a good quality of life without further treatment.

## Discussion

This article reports on the neurological complications of a female patient with severe aplastic anemia before and after haplo-HSCT. Haplo-HSCT is a primary and effective treatment for patients with hematological disorders, but it is often associated with a range of complications, including neurological involvement (14). During the entire diagnosis and treatment process, the patient had multiple neurological symptoms, which could be explained by PRES, and CNS-GVHD respectively. Therefore, CNS-GVHD was regarded as the dominant pathophysiological process in this case. According to the diagnostic criteria specified in the clinical practice consensus of cGVHD in 2010 (6, 15), this patient met the following criteria: The patient had neurological symptoms, cGVHD affecting other organs, and CNS involvement without other causes; MRI showed neurological lesions; The patient responded to immunosuppressive treatment. Therefore, a diagnosis of CNS-GVHD was considered the most likely diagnosis. According to the 2023 Lancet Neurology consensus on MOGAD, diagnosis requires three criteria: First, the patient must present with at least one typical central nervous system demyelinating event. Second, a highly sensitive and specific live cell-based assay should be used, providing both qualitative and quantitative results; clear positivity confirms the diagnosis, while low titer or undetermined results require confirmation with clinical and imaging features. Lastly, other potential diagnoses, such as multiple sclerosis, must be excluded (16). In our patient, the overall clinical, radiological, and laboratory profile did not fully meet these criteria, particularly in view of the atypical features and weak, assay-dependent MOG-IgG signal; therefore, she cannot be classified as having definite MOGAD.

To better integrate the clinical manifestations of this case within an immunopathological framework, it is essential to consider the mechanistic convergence between CNS-GVHD and MOGAD. Although these two entities arise in distinct clinical contexts, both represent aberrant immune responses targeting CNS antigens, with substantial overlap in downstream effector pathways (6).CNS-GVHD is characterized predominantly by perivascular CD8^+^ cytotoxic T-cell infiltration, microglial activation, endothelial injury, and BBB disruption driven by donor-derived alloreactive lymphocytes, creating a pro-inflammatory milieu enriched in IFN-γ, TNF-α, GM-CSF, and chemokines that promote leukocyte trafficking across the BBB and may prime the CNS for secondary autoimmune processes. By contrast, MOGAD is driven by pathogenic IgG1 antibodies against MOG, leading to antibody-dependent cellular cytotoxicity, complement activation, and demyelination (17, 18). Importantly, MOG-IgG seroconversion generally requires CNS antigen exposure; thus, conditions that disrupt the BBB may facilitate the release or unmasking of MOG epitopes (17, 19). In this patient, the temporal sequence of severe PRES followed by cGVHD-related immune dysregulation likely created a permissive landscape for aberrant antigen presentation and subsequent MOG antibody production. Shared pathogenic themes, including BBB dysfunction, T cell–mediated tissue injury, complement activation, and innate immune activation, provide a plausible immunopathological framework for understanding how CNS-GVHD could be accompanied by a transient MOG-directed humoral response (20, 21).

In this patient, two temporally distinct neurological phases were observed. An early episode consistent with PRES occurred prior to the onset of systemic GVHD and was considered more likely related to calcineurin inhibitor–associated neurotoxicity. This episode improved after adjustment of immunosuppressive therapy. Importantly, subsequent progressive neurological manifestations emerged after the development of systemic GVHD, and were accompanied by evolving inflammatory MRI lesions and a favorable response to immunomodulatory therapy, supporting an immune-mediated process consistent with CNS-GVHD. A study by Wieczorek et al. revealed that 57.9% of allo-HSCT-associated neurological complications are immune-mediated (22). We align with this perspective in our case. Specifically, we favor an interpretation of predominantly immune-mediated CNS-GVHD with a secondary MOG-directed humoral response, rather than primary MOGAD. The patient’s specific seropositivity for MOG antibodies may be linked to her prior history of PRES. Since MOG is a key component of myelin sheaths (23), its antibody production requires antigen exposure within the nervous system (24, 25). Zhang et al. (14)likewise propose that MOG antibodies may mediate demyelinating damage through direct attack on the myelin sheath or activation of the complement system. Therefore, we hypothesize that this patient’s MOG seroconversion resulted from combined effects of PRES-induced BBB hyperpermeability and dysfunctional immune regulation during cGVHD, enabling aberrant immune recognition of MOG antigens.

The incidence of CNS-cGVHD is lower than other common target organs. Due to limited reported cases, its clinical features, treatment response, and prognosis remain unclear, making diagnosis and management challenging (26, 27). In a multicenter cohort of 66 patients with CNS manifestations in the setting of acute or chronic GVHD, Lambert et al. reported that cognitive and behavioral changes, sensory disturbances, and gait imbalance were the most frequent presentations, with MRI abnormalities in more than half of cases and corticosteroids as the mainstay of therapy (28). Other observational studies and case series similarly describe heterogeneous phenotypes occurring on a background of systemic cGVHD and often responding to intensified immunosuppression (29, 30). Immune-mediated neuropathies and CNS inflammatory disorders, including myelitis and encephalitis, have been increasingly recognized after allo-HSCT and are often associated with, or preceded by, cGVHD, supporting the concept that chronic alloimmune activation can trigger secondary, autoantibody-mediated complications (31, 32). Yokoyama et al. (33) reported a patient who developed progressive paraplegia and sensory disturbance after allo-HSCT, with MRI findings and a clinical course resembling multiple sclerosis. The disease progressed despite high-dose corticosteroid therapy but improved after treatment with rituximab, suggesting that B cell–mediated or antibody-related mechanisms may contribute to a subset of CNS-cGVHD–associated myelitis. Hümmert et al. (34)described severe allo-immune antibody–associated peripheral and central nervous system disease after allo-HSCT, with detection of neural autoantibodies and responsiveness to immunotherapy, illustrating that donor–host immune conflict may evolve into highly targeted humoral autoimmunity. Comparison with idiopathic MOGAD also informs interpretation of our findings. Large contemporary cohorts indicate that MOGAD typically affects children and young adults, with optic neuritis, transverse myelitis, brainstem syndromes, and cortical encephalitis as dominant phenotypes, and a substantial proportion following a relapsing course (35, 36).

Currently, detection techniques such as ELISA used in clinical practice can hardly identify patients with MOGAD (37). Although CBA and TBA are commonly used methods (38, 39), the live cell-based assay, as per the 2023 Lancet Neurology consensus on MOGAD (16), offers higher sensitivity and specificity. In this case, routine CBA and TBA tests were negative, but a weakly positive result was obtained through live cell immunofluorescence (1:32 titer). It is important to note that weakly positive results can be affected by poor signal-to-noise differentiation, experimental conditions, and nonspecific binding, leading to false positives. Additionally, conditions like PRES or cGVHD-related immune dysregulation may trigger low-titer MOG antibodies, a phenomenon known as the “bystander effect.” Therefore, weak positives should be interpreted with clinical and imaging features to avoid misdiagnosis, and follow-up titers or repeat testing is recommended for confirmation. At present, there is insufficient clinical, radiological, and immunological evidence to support a causal relationship between the myelitis and MOG antibodies. Though not widely used yet, this method will become increasingly important in MOG antibody detection with advances in technology and reduced costs (18). Conventional fixed-cell CBAs and tissue-based assays may under-detect MOG-IgG, because fixation can alternative MOG conformation or mask extracellular epitopes, reducing antibody binding especially when titers are low. In contrast, live cell–based assays preserve the native membrane topology of MOG, offering substantially greater sensitivity for weak or transient antibody responses (40). Indeed, in a large real-world cohort, 56 of 594 paired sera tested positive only by live-cell CBA, while fixed CBA remained negative (41). Given that our patient had low-titer, rapidly reversible MOG-IgG, the negative conventional CBA/TBA results likely reflect a combination of methodological insensitivity and the biologically limited magnitude of the MOG-specific response, rather than absence of autoantibody.

CNS-GVHD has long been considered rare and diagnostically challenging. However, accumulating evidence indicates that immune-mediated inflammatory disorders of the brain and spinal cord occurring after allogeneic hematopoietic stem cell transplantation should be included among the key differential diagnoses once infection, drug-related neurotoxicity, vascular events, and relapse of the underlying disease have been carefully excluded (28, 42). Clinically, CNS-GVHD is characterized by marked heterogeneity. The most common presentation is an encephalopathy-dominant syndrome featuring cognitive decline, altered consciousness, seizures, and neuropsychiatric symptoms, while focal neurological deficits, myelopathy, and cerebellar syndromes are also observed (28, 29). Notably, the timing of CNS-GVHD does not strictly conform to the conventional day-100 distinction between acute and chronic GVHD; instead, neurological involvement may occur both early and late after transplantation and is frequently temporally associated with tapering or withdrawal of immunosuppressive therapy. From a neuroimaging perspective, CNS-GVHD lacks disease-specific MRI features. Reported abnormalities commonly include multifocal or diffuse white-matter hyperintensities with demyelination-like patterns, vascular or microangiopathic changes, and encephalitic involvement of cortical, limbic, or cerebellar regions (34, 43, 44). Accordingly, MRI primarily serves to support the presence of an inflammatory or immune-mediated process and to exclude alternative etiologies rather than to establish a definitive diagnosis. CSF examination may reveal mild pleocytosis, elevated protein levels, or evidence of intrathecal immunoglobulin synthesis, but these findings are nonspecific; their principal value lies in excluding central nervous system infection and providing ancillary support for an immune-mediated mechanism (21). Although neuronal or glial autoantibodies have been detected in a small number of post-transplant patients with neurological complications, there is currently no evidence that such antibodies represent specific biomarkers of CNS-GVHD. Instead, they more likely reflect concomitant autoimmune central nervous system inflammation in the setting of post-transplant immune dysregulation (45). Conversely, a negative autoimmune encephalitis antibody panel does not preclude the diagnosis of CNS-GVHD, as available data suggest that its pathogenesis is predominantly driven by T cell– and microglia-mediated cellular immune inflammatory responses (46).

With respect to treatment, systemic corticosteroids in combination with optimization of baseline immunosuppression remain the first-line therapeutic approach, although responses are variable and relapses are not uncommon. For steroid-refractory or relapsing cases, immunomodulatory therapies such as rituximab may be considered (47). After empirical treatment with corticosteroids for the patient, live-cell immunofluorescence detection was performed again, and the MOG antibody turned negative. Existing literature shows that approximately 25% of MOGAD patients become seronegative after treatment (8), accompanied by the improvement of the patient’s neurological symptoms. The temporal association between immunotherapy, seroconversion to MOG-IgG negativity, and clinical improvement suggests that the humoral response may have contributed to symptom expression, but it does not prove that MOG antibodies were the primary pathogenic driver, particularly in the presence of concomitant CNS-GVHD. Furthermore, negative results from conventional testing may be partially attributable to the patient’s milder central neurological manifestations, compounded by pretreatment with corticosteroids and IVIG prior to assay conduction. However, no studies have established a correlation between MOG antibody titers and disease severity. Regarding persistently positive MOG antibodies, current evidence more strongly associates this finding with relapse in MOG antibody-associated disorders. The patient exhibited a fluctuating neurological course, and partial clinical improvement was observed in parallel with immunomodulatory therapy administered for systemic GVHD. This temporal association further supports an immune-mediated mechanism underlying the neurological manifestations and provides additional indirect evidence for CNS-GVHD. Zhang et al. (14) observed that in six cases of MOG-associated disorders, CSF IgG synthesis rates modestly decreased post-treatment, yet MOG antibody levels failed to decline correspondingly. One patient relapsed after 4 months, suggesting that while immunosuppressive therapy partially controlled intrathecal inflammation, the persistence of MOG antibodies remained predictive of recurrence risk. Nevertheless, a significant proportion of seropositive patients do not experience disease recurrence (48, 49).

We consider the transient MOG seropositivity observed in this case to be more consistent with a bystander phenomenon in the context of central nervous system GVHD, as the typical clinical and radiological features of classical MOGAD were not observed. However, the identification of low-titer MOG-IgG solely by a highly sensitive live cell–based assay remains a hypothesis-generating observation intended to provide clinical experience. Additional post-HSCT cases are required to determine whether this phenotype represents a reproducible manifestation related to CNS-GVHD. MOG testing was considered only in the presence of a delayed, subacute immune-mediated neurological phenotype. Based on this experience, we do not recommend routine MOG screening in early toxic-metabolic encephalopathy or in typical post-HSCT PRES, but suggest considering MOG-IgG testing when the clinical course becomes atypical. Overall, current consensus supports that CNS-GVHD should be regarded as a “possible” or “probable” diagnosis based on an integrated assessment of clinical manifestations, neuroimaging findings, laboratory results, and response to immunomodulatory therapy (28), rather than on any single disease-specific test, underscoring the need for standardized diagnostic criteria and prospective studies.

## Conclusion

This case report describes low-titer MOG antibody seroconversion detected only by live-cell immunofluorescence in an allogeneic HSCT recipient with CNS-cGVHD and prior PRES. It illustrates that highly sensitive live-cell assays may reveal transient MOG-directed autoreactivity that is not captured by conventional CBA/TBA, but such findings in isolation should be interpreted cautiously and not regarded as definitive evidence of MOG antibody–mediated disease. Our observations support considering MOG-IgG testing, including live-cell–based methods, in selected HSCT recipients with unexplained or atypical CNS inflammation and active cGVHD after exclusion of other causes, while recognizing CNS-GVHD as the dominant pathology. Future studies are needed to determine how frequently post-transplant MOG autoreactivity occurs, to clarify its clinical significance, and to assess whether it identifies patients who might benefit from tailored immunomodulatory strategies.

## Data Availability

The original contributions presented in the study are included in the article/**Supplementary Material**. Further inquiries can be directed to the corresponding author.
