# Supplementary figures and images for: Neurological graft-versus-host disease with MOG antibody positivity after allogeneic stem cell transplantation: a case report
Source: Front Immunol. 2026 Mar 11;17:1684838. doi: 10.3389/fimmu.2026.1684838 (PMC13016198; doi:10.3389/fimmu.2026.1684838)

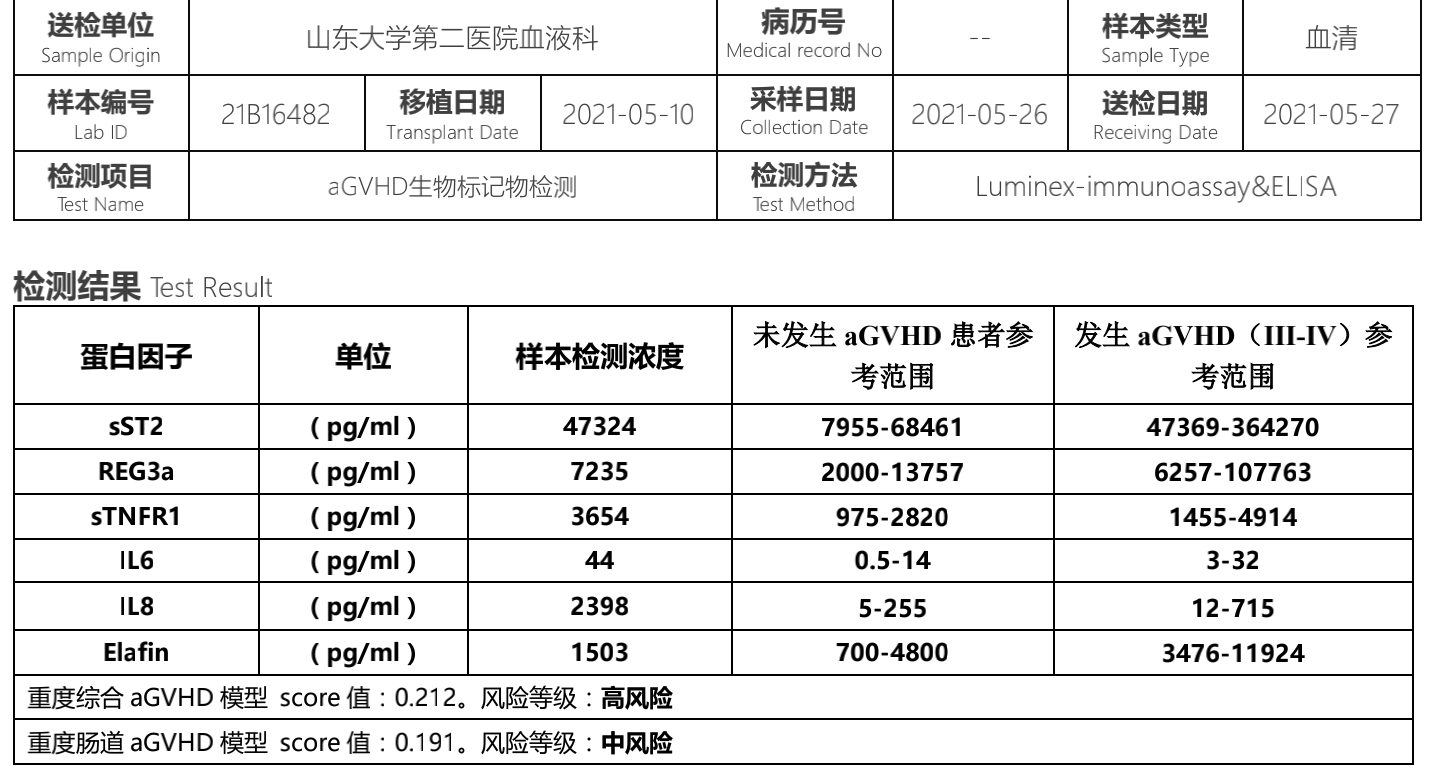

Supplement: Supplementary file 1 [file Image1.tiff]

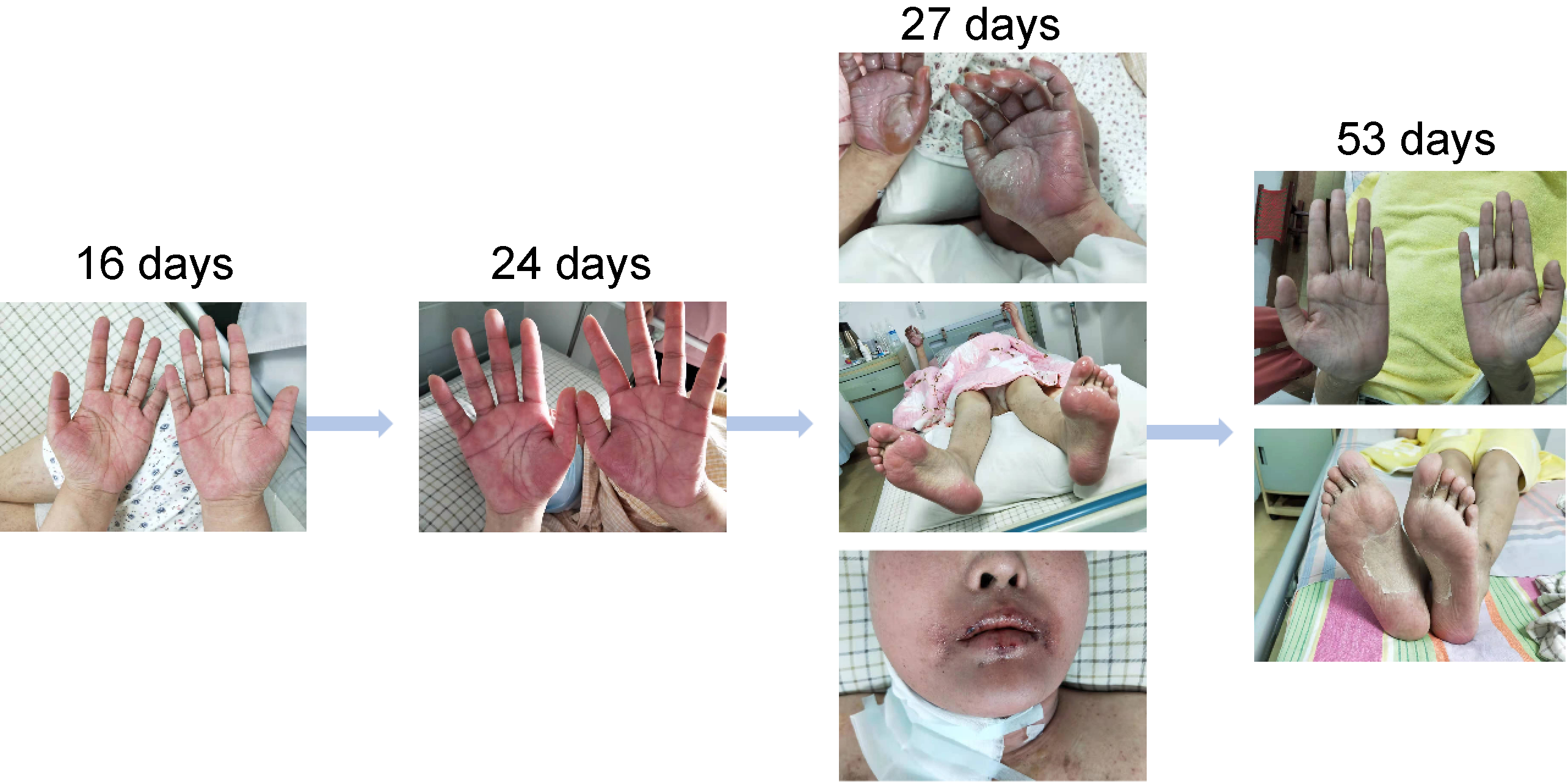

Supplement: Supplementary file 2 [file Image2.tiff]

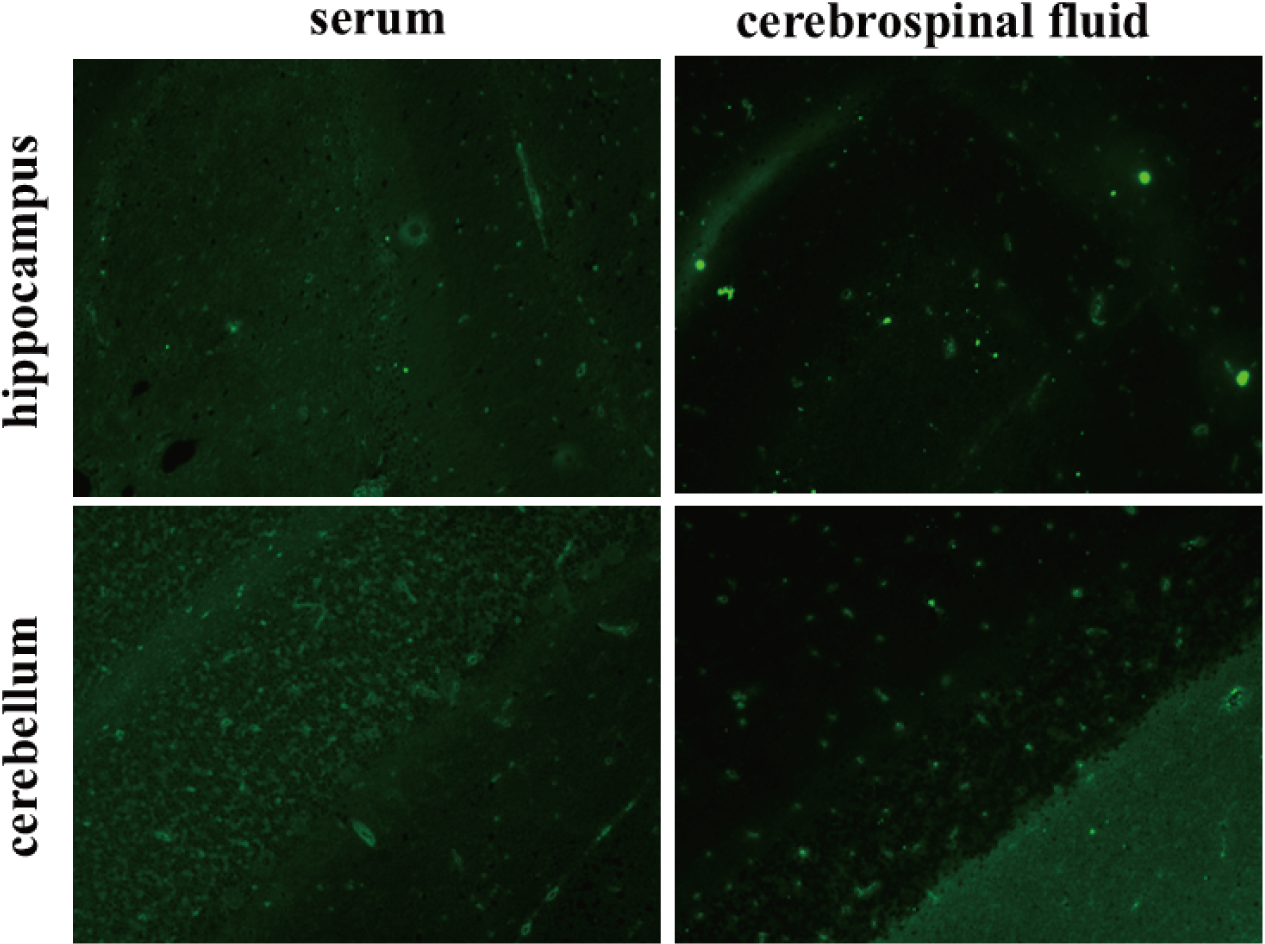

Supplement: Supplementary file 3 [file Image3.tif]

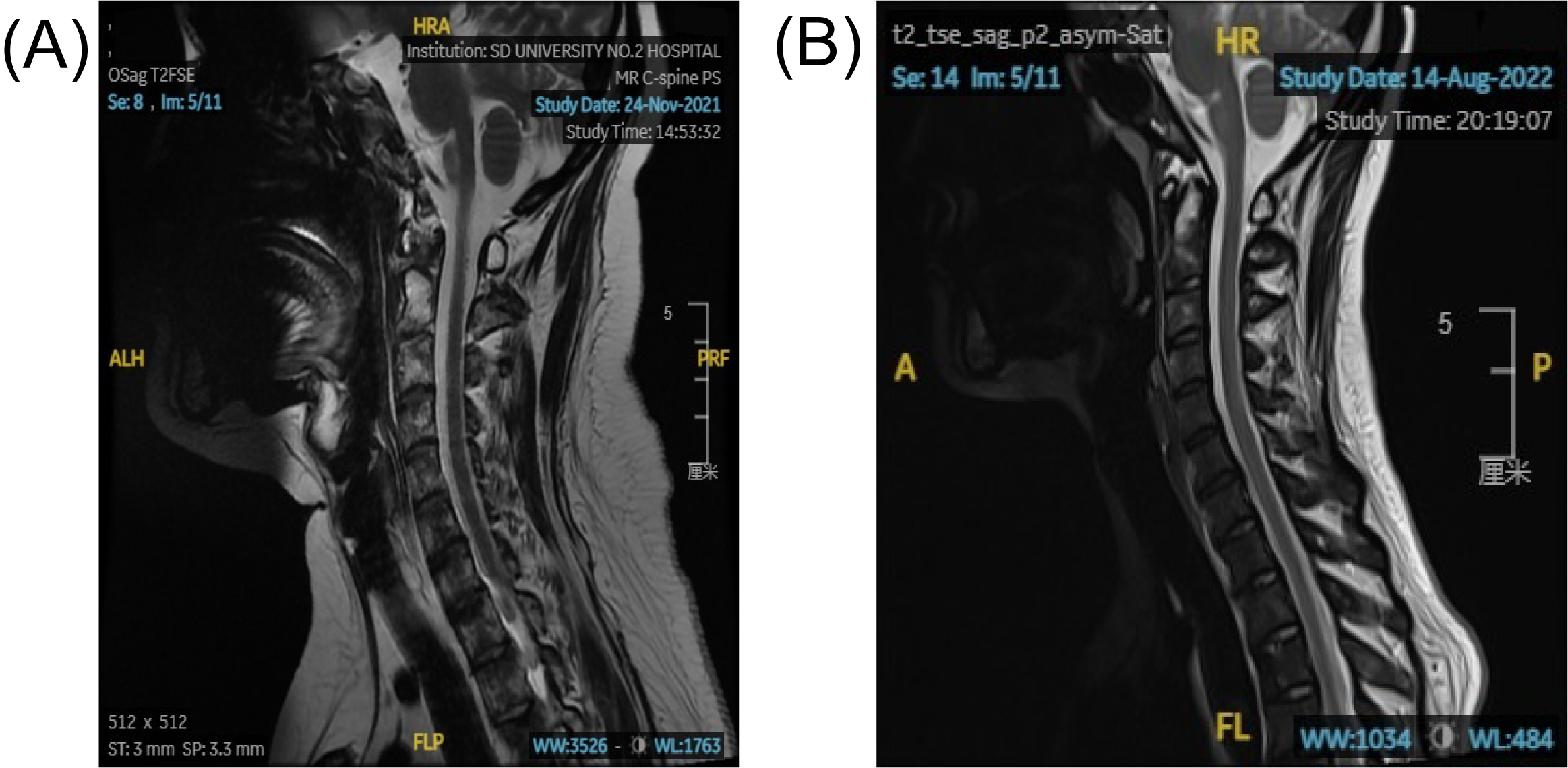

Supplement: Supplementary file 4 [file Image4.tif]
