# Supplementary material for: Neurological graft-versus-host disease with MOG antibody positivity after allogeneic stem cell transplantation: a case report
Source: Front Immunol. 2026 Mar 11;17:1684838. doi: 10.3389/fimmu.2026.1684838 (PMC13016198; doi:10.3389/fimmu.2026.1684838)
Supplement: Supplementary file 5 [file Table1.docx]

Supplementary Material

**The detailed procedure for the live cell–based assay is as follows:**

1. Sample pre-treatment

1.1 Blood samples

Peripheral blood was collected and centrifuged at 2,000 rpm for 10 min. The upper serum layer was carefully transferred into a clean 1.5 mL microcentrifuge tube and labeled with the sample ID. Serum or plasma samples were stored at −20°C or below until testing.

1.2 Cerebrospinal fluid (CSF)

CSF samples were collected according to standard clinical procedures and used undiluted for the assay. Samples were stored at −20°C or below until testing.

2. Reagent preparation (prepared fresh as needed)

2.1 PBS solution

One package of PBS powder (sufficient for 1,000 mL) was dissolved in 1,000 mL of double-distilled water (ddH₂O). After complete dissolution, 2 mL Tween-20 was added to prepare PBS containing 0.2% Tween-20, and the solution was mixed thoroughly.

2.2 Blocking solution (Mixture A)

Goat serum was diluted with PBS to obtain a solution containing 10% goat serum (v/v), referred to as Mixture A.

2.3 Secondary antibody solution (Mixture B)

The fluorescently labeled secondary antibody was diluted 1:500 in Mixture A to prepare Mixture B immediately before use.

3. Antibody detection

3.1 Plate preparation

The antigen-coated plate was removed from storage. Any residual PBS in the wells was discarded. Then, 100 µL PBS was added to each well for one wash, and the supernatant was discarded.

3.2 Primary incubation

For serum/plasma and control wells, 80 µL of Mixture A was added, followed by 4 µL of patient serum/plasma, negative control, or positive control. CSF samples did not require dilution; 80 µL of CSF was added directly to the wells. The plate was gently shaken to mix and incubated at 37°C for 30 min (or 1 h at room temperature).

3.3 Washing and fixation

After incubation, the liquid was discarded, and each well was washed three times with 100 µL PBS on a shaker (speed 100) for 3 min per wash. Fixative was then added according to the manufacturer’s instructions to fix the cells, followed by three additional washes with 100 µL PBS (3 min per wash with gentle rocking).

3.4 Secondary incubation

After removal of the wash buffer, 80 µL of Mixture B was added to each well. The plate was gently shaken to mix, covered with aluminum foil, and incubated in the dark at 37°C for 30 min (or 1 h at room temperature).

3.5 Final washing and microscopy

After incubation with the secondary antibody, the liquid was discarded and each well was washed three times with 100 µL PBS (3 min per wash with gentle rocking on a shaker at speed 100). Finally, 100 µL PBS was added to each well to cover the cells. The plate was examined under a fluorescence microscope, and representative images were acquired.

4. Interpretation and positivity criteria

First, the green channel was examined to confirm successful plasmid transfection (transfected cells showing green fluorescence). The red channel was then evaluated for human IgG binding.

**Positive:** If transfected cells in the sample well showed distinct red fluorescence predominantly along the cell membrane (with non-transfected cells showing no or only minimal red fluorescence, or markedly weaker signal than transfected cells), and the cell membrane outline was clearly delineated, the sample was judged positive for the antibody. **Negative:** If transfected cells in the sample well showed no obvious red fluorescence on the cell membrane or within the cell, or if red fluorescence lacked clear membrane localization, or if non-transfected cells displayed comparable red fluorescence, or if transfection failed (absence of green fluorescence), the sample was judged negative.

**2.Supplementary Figures**

**
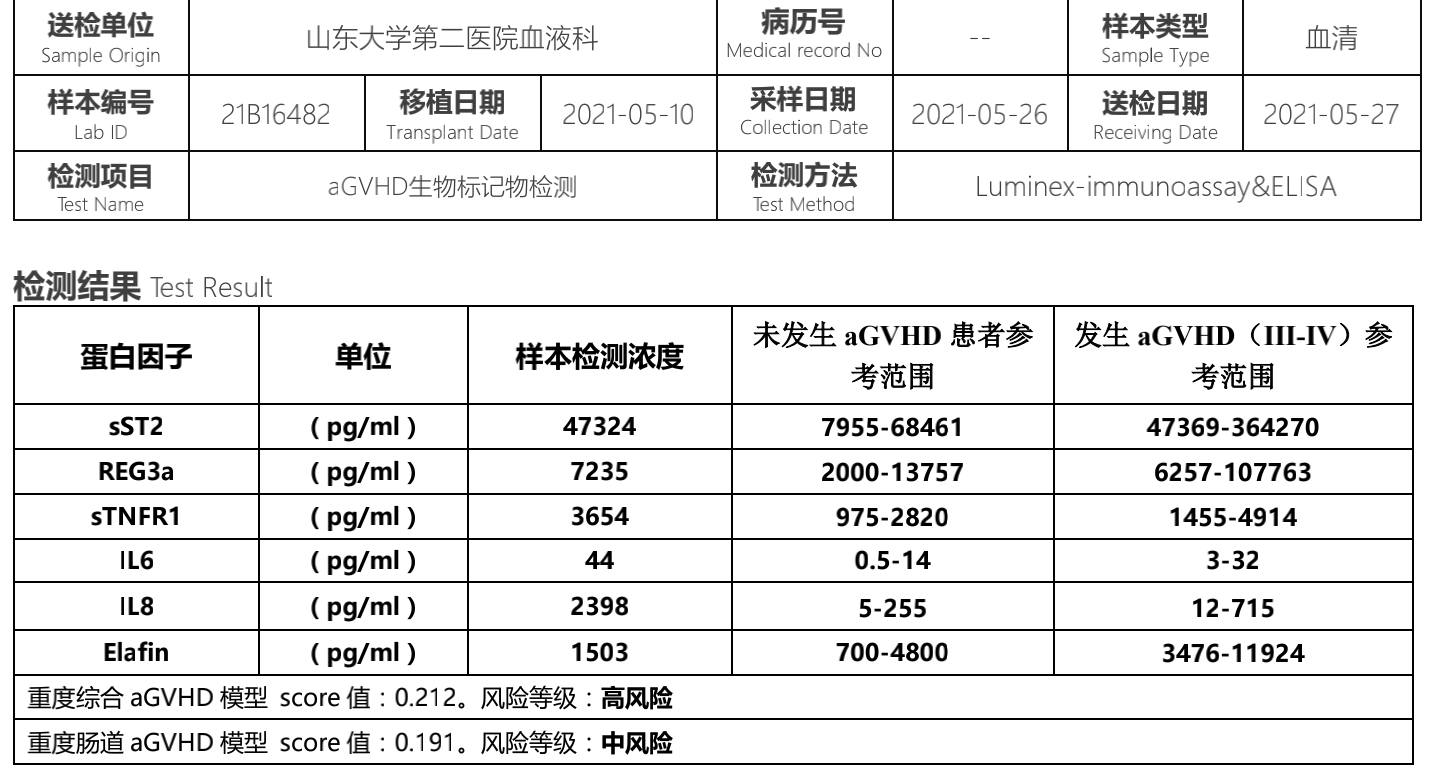
Figure S1.** aGVHD Biomarker Detection Results


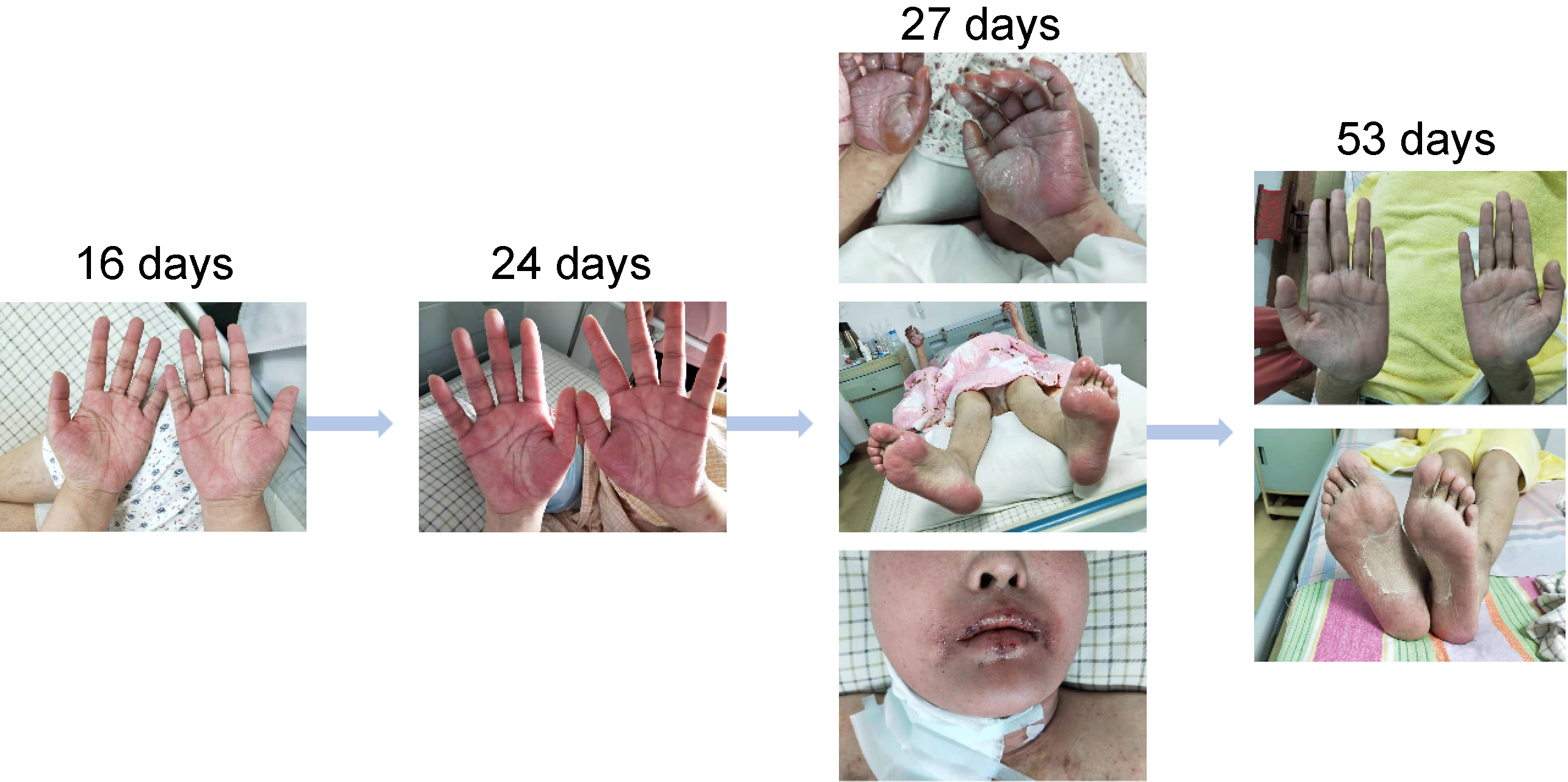


**Figure S2. Skin manifestations of GVHD after Haplo-HSCT treatment.** This figure shows the evolution of cutaneous aGVHD following haploidentical HSCT. The patient initially developed erythematous maculopapular lesions involving the face, neck, chest, back, and palms (grade I), which subsequently progressed to confluent erythema with localized vesicle formation (grade II).


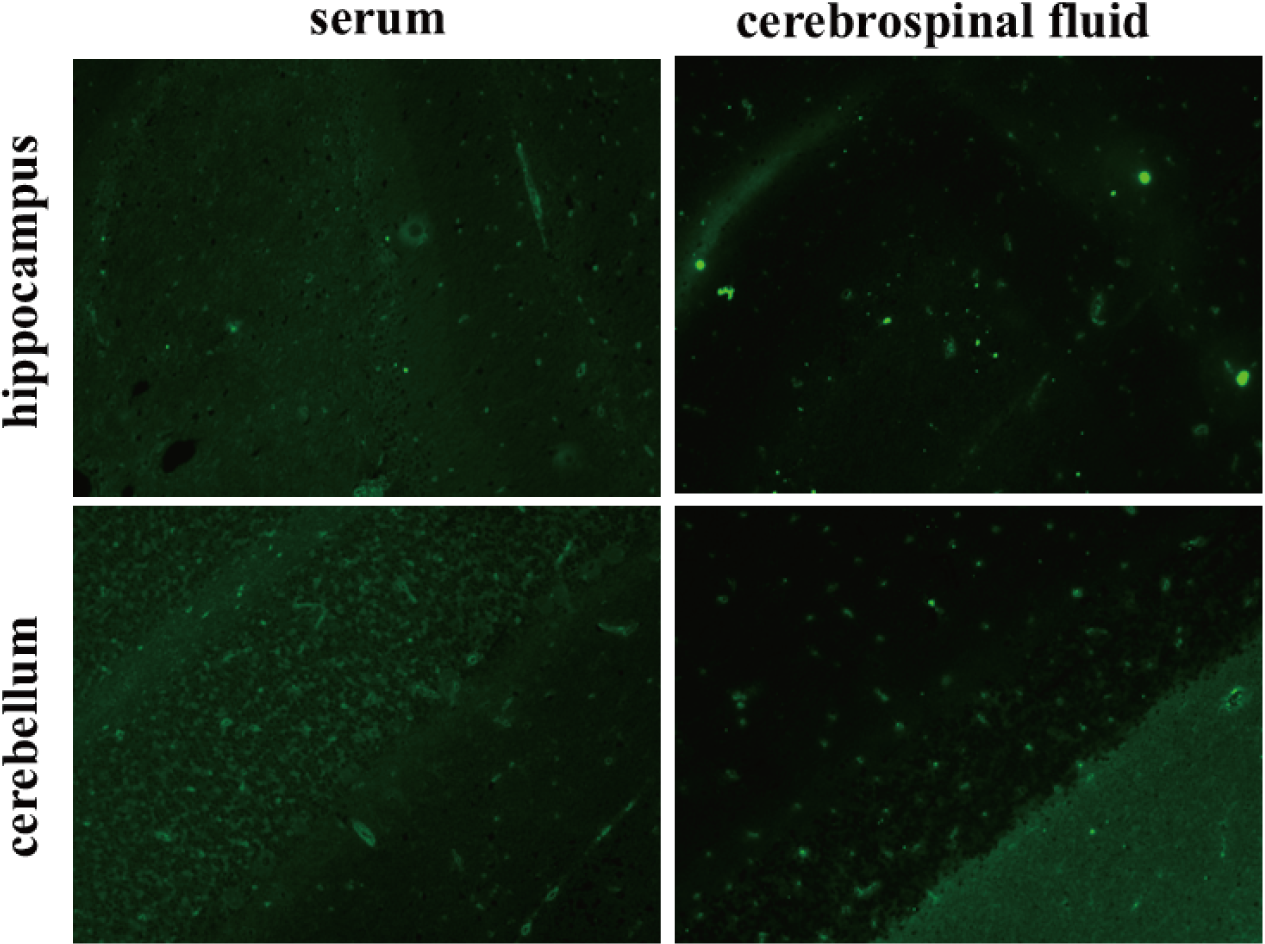


**Figure S3.** CBA and TBA demonstrated uniformly negative results for MOG-IgG in CSF and serum when tested against brain tissue sections from distinct neuroanatomical regions.


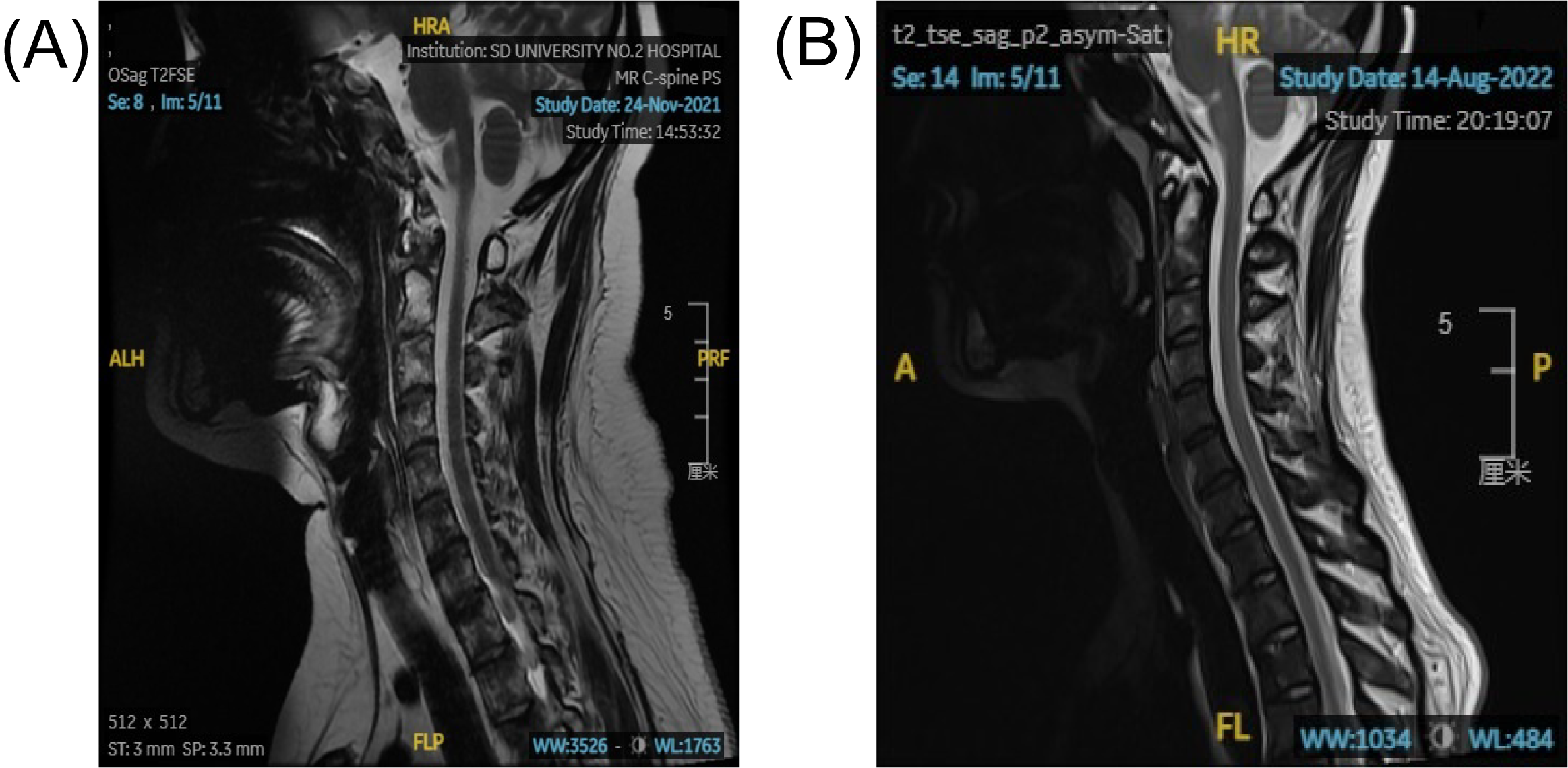


**Figure S4. (A)** Sagittal T2-weighted cervical spinal MRI obtained on November 24, 2021 demonstrating scattered patchy hyperintense lesions within the cervical spinal cord with reduced extent compared with prior imaging, consistent with interval radiological improvement. **(B)** Sagittal T2-weighted cervical spinal MRI obtained on August 14, 2022 showing no definite focal abnormal signal within the cervical spinal cord, consistent with stable to improved spinal cord findings compared with prior imaging.
